# Human Helminth Co-Infection: Analysis of Spatial Patterns and Risk Factors in a Brazilian Community

**DOI:** 10.1371/journal.pntd.0000352

**Published:** 2008-12-23

**Authors:** Rachel L. Pullan, Jeffrey M. Bethony, Stefan M. Geiger, Bonnie Cundill, Rodrigo Correa-Oliveira, Rupert J. Quinnell, Simon Brooker

**Affiliations:** 1 London School of Hygiene and Tropical Medicine, London, United Kingdom; 2 René Rachou Research Centre FIOCRUZ, Belo Horizonte, Brazil; 3 The George Washington University, Washington D.C., United States of America; 4 University of Leeds, Leeds, United Kingdom; The University of Queensland, Australia

## Abstract

**Background:**

Individuals living in areas endemic for helminths are commonly infected with multiple species. Despite increasing emphasis given to the potential health impacts of polyparasitism, few studies have investigated the relative importance of household and environmental factors on the risk of helminth co-infection. Here, we present an investigation of exposure-related risk factors as sources of heterogeneity in the distribution of co-infection with *Necator americanus* and *Schistosoma mansoni* in a region of southeastern Brazil.

**Methodology:**

Cross-sectional parasitological and socio-economic data from a community-based household survey were combined with remotely sensed environmental data using a geographical information system. Geo-statistical methods were used to explore patterns of mono- and co-infection with *N. americanus* and *S. mansoni* in the region. Bayesian hierarchical models were then developed to identify risk factors for mono- and co-infection in relation to community-based survey data to assess their roles in explaining observed heterogeneity in mono and co-infection with these two helminth species.

**Principal Findings:**

The majority of individuals had *N. americanus* (71.1%) and/or *S. mansoni* (50.3%) infection; 41.0% of individuals were co-infected with both helminths. Prevalence of co-infection with these two species varied substantially across the study area, and there was strong evidence of household clustering. Hierarchical multinomial models demonstrated that relative socio-economic status, household crowding, living in the eastern watershed and high Normalized Difference Vegetation Index (NDVI) were significantly associated with *N. americanus* and *S. mansoni* co-infection. These risk factors could, however, only account for an estimated 32% of variability between households.

**Conclusions:**

Our results demonstrate that variability in risk of *N. americanus* and *S. mansoni* co-infection between households cannot be entirely explained by exposure-related risk factors, emphasizing the possible role of other household factors in the heterogeneous distribution of helminth co-infection. Untangling the relative contribution of intrinsic host factors from household and environmental determinants therefore remains critical to our understanding of helminth epidemiology.

## Author Summary

Helminth species such as *Necator americanus* and *Schistosoma mansoni* are among the most prevalent of chronic human infections in the developing world. Individuals living in endemic areas are commonly infected with both species. Although the implications of being co-infected with helminths are increasingly recognized, factors influencing patterns of co-infection within human communities remain ill-defined. Here, we describe spatial patterns and risk factors for co-infection with *N. americanus* and *S. mansoni* in a co-endemic area in south-eastern Brazil. The prevalence of co-infection with these two helminths in this region was high (41%), varied across the study area and was clustered in high-risk households. We reveal that factors associated with lower socio-economic status (relative socio-economic status, household crowding) and residential environment (living in the eastern watershed or in areas with less vegetation) were significantly associated with the risk of co-infection relative to being uninfected with either species. Importantly, much of the variability in risk between households (i.e. household clustering) could not be readily explained by these risk factors. The results suggest that, whilst measures aimed at reducing exposure to infection may have an important impact on co-infection and its associated morbidity, untangling the relative contribution of intrinsic host factors (e.g. immune response) from household and environmental determinants remains critical to our understanding of helminth epidemiology.

## Introduction

People living in poor areas of the tropics commonly harbour multiple parasitic infections, including infection with multiple helminth species [1],[2]. An increasing number of studies demonstrate that individuals infected with multiple helminth species tend to harbour the most intense infections [3]–[11] and can be at an increased risk of infection-related morbidity [12]–[15]. For example, a study of Brazilian school children showed those harbouring concomitant infection with *Ascaris lumbricoides* and *Trichuris trichiura* were at increased risk of stunting [16], whilst another Brazilian study found the risk of anaemia among school children infected with *Schistosoma mansoni* and two or three soil-transmitted helminth (STH) infections was significantly higher that those harbouring single STH species [12]. The occurrence of extensive polyparasitism in human communities also has important implications for a multiple infection approach to control [17].

Recent interest in the scientific study of polyparasitism has given renewed prominence to some old epidemiological questions; in particular identifying factors governing patterns of infection. A wealth of epidemiological investigation across numerous ecological and socio-economic settings indicate that certain characteristics are common to the epidemiology of single helminth species in communities, including household clustering and spatial heterogeneity [18]. Such features most likely result from the combined effects of extrinsic (exposure to infection) and intrinsic (host resistance) factors [18],[19]. However, our understanding of the determinants of multiple helminth species infection patterns within communities remains poorly defined. For example, while recent studies have documented the prevalence of multiple helminth infections and their patterns by age and sex [3]–[10], little is known about spatial and household clustering of multiple helminth infection within communities or putative risk factors [20].

In the paper, we investigate the spatial patterns and household clustering of helminth co-infection and associated risk factors among individuals living in the state of Minas Gerais, Brazil. A previous analysis has already highlighted the high frequency of multiple helminth infection in the area [21]. Although *A. lumbricoides* is also endemic to the region, we focus specifically on co-infection with the hookworm *Necator americanus* and *S. mansoni* since these species both contribute to iron-deficiency anaemia (via distinct mechanisms [22],[23]) but have dissimilar life cycles and modes of transmission. First, we explore spatial patterns of co-infection with *N. americanus* and *S. mansoni* using spatial statistics. We then investigate the role of individual, household and environmental risk factors in explaining the observed heterogeneities in infection patterns using a multi-level Bayesian multinomial approach, whereby individuals are assumed to be clustered within households. This approach permits robust, unbiased investigation of within-household clustering.

## Materials and Methods

### Study area and procedures

**Figure 1:**
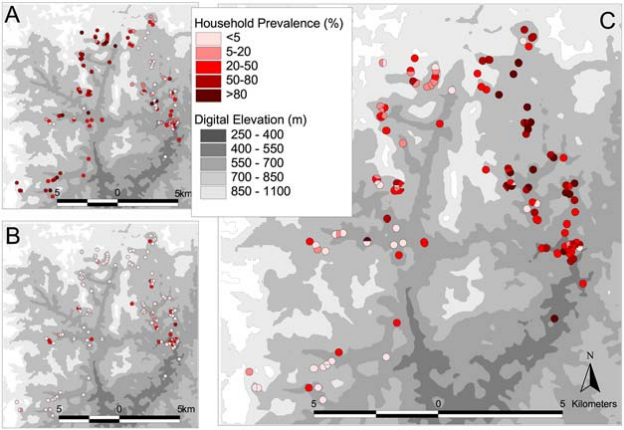
Map of Americaninhas, Minas Gerias State. A Location of the study area in Minas Gerias State; B distribution of households within the study area (urban municipality inset).

The study was conducted from June to September 2004 in Americaninhas, a region in the municipality of Nova Oriente in the northeast of Minas Gerias state, which is situated in southeast Brazil (Figure 1). Details of the study area, recruitment method, and cross-sectional parasitological and questionnaire surveys have been provided elsewhere [21],[24],[25] and only a summary is given here. The area is hilly and has an average temperature of 24°C, with a rainy season between November and March; annual rainfall is 1300–2000 mm. The study area is divided by a high ridge of land running north-south, separating the study area into two distinct zones or watersheds. The majority of inhabitants are involved in rural subsistence farming; cattle ranching is another important source of income.

A series of meetings was held with community members to explain the purpose of the study. that participation was voluntary and that participants were able to withdraw from the study at any time. Written or oral consent was obtained from all adult subjects and from parents or guardians of minors. A pre-tested standardized questionnaire was administered to the head of each household to collect information on household socio-economic characteristics including house construction, water and sanitation, parental education, and ownership of selected household assets. During the parasitological survey, stool samples were collected over the course of two days (if possible) and were initially examined using the formalin-ether sedimentation technique for the presence of helminth eggs. Individuals positive for any helminth infection were subsequently examined by the Kato–Katz faecal thick smear technique to quantify the intensity of the infection expressed as eggs per gram of faeces (epg). Two slides were taken from each day's faecal sample for a total of up to four slides from each individual. Morphological examination of expelled worms following treatment among a sub-sample of individuals showed that hookworm infection was exclusively of the species *Necator americanus* [25]. A polymerase-chain-reaction (PCR) test was performed on 20 of the above samples to confirm the morphological examination as *Necator americanus* [26]. *N. americanus* was found in 100% of these samples, no *A. duodenale* infection was found.

Household locations were mapped using a hand-held Trimble GeoExplorer global positioning system (GPS) receiver (Trimble Navigation, Sunnyvale, CA, USA) and ArcPad 6.0.3 software (Environmental Systems Research Institute Inc., Redlands, CA, USA). Readings, with a resolution of 5 m, were taken at the front door, or as near as possible in order to receive a sufficient satellite reception and an average of 10 readings of the co-ordinates were taken. Remotely sensed proxy environmental data were extracted for May 2001 from the Advanced Spaceborne Thermal Emission and Reflection Radiometer (ASTER) satellite sensor at 30 m spatial resolution (http://edcdaac.usgs.gov/aster/asterdataprod.asp). ASTER provides information on Normalized Difference Vegetation Index (NDVI), a proxy of vegetation density and soil moisture, and digital elevation [27]. The GIS was compiled and all maps were created using ArcView 3.3 (Environmental Systems Research Institute Inc., Redlands, CA, USA).

The study was reviewed and approved by the ethical committee of the Centro de Pesquisas René Rachou-FIOCRUZ and the Brazilian National Committee for Ethics in Research (CONEP), and the ethical review boards of George Washington University (USA) and London School of Hygiene and Tropical Medicine (UK). Individuals found to be infected with any soil transmitted helminth or with *S. mansoni* were treated with a single dose of 400 mg albendazole and 40 mg/kg praziquantel, respectively.

### Data analysis

Participants were recorded as positive for an infection with *S. mansoni* or *N. americanus* if at least one egg was detected by either formalin-ether sedimentation or Kato–Katz faecal thick smear. Participants were classified into five age-groups: under 5 years, younger children (5–9 years), older children (10–19 years), adults (20–59 years) and over 60 years. Information on ownership of household assets was used to construct a wealth index using principal component analysis, using the method of Filmer and Pritchett [28]. Following this approach, households were divided into tertiles, to provide a categorical measure of relative socio-economic status. Household factors potentially directly associated with infection outcomes (such as toilet facilities and household construction) were not included in the wealth index to allow for independent assessment of their involvement: details of the derived wealth index are provided elsewhere [25]. Information from the digital elevation model was used to divide households into either the eastern or western watershed. Housing density calculated in ArcView 3.3 was used to categorise households as urban (>55 households within 1 km of the household), rural (5–55 households within 1 km) and isolated (<5 households within 1 km), with cut-offs chosen to reflect the distribution of households within the study region.

As an outcome measure a (mutually exclusive) multi-categorical response for infection status was constructed as follows: (i) no infection, (ii) mono-infection with *N. americanus*, (iii) mono-infection with *S. mansoni* and (iv) co-infection with *N. americanus* and *S. mansoni*. In order to assess the importance of demographic, socio-economic and environmental risk factors on the occurrence of mono- and co-infection simultaneously we used a multinomial modelling approach, which extends logistic regression by estimating the effects of explanatory variables on the probability that the outcome is in a particular category. Initially, for each covariate frequentist unadjusted multinomial models were fit on the outcome in Stata 9.1 (College Station Texas, USA), and covariates with *P*>0.2 (Wald test) were excluded from further analysis. Standard errors were adjusted for dependence between individuals within households. Scatter-plots and the entry of categorised predictor variables were used to investigate non-linear relationships.

Subsequently, the retained covariates were built into a Bayesian multinomial mixed effect model in WinBUGS Version 14 (MRC Biostatistics Unit, Cambridge, UK). To account for dependence of individuals within households, household was included as a random effect. We employed a Bayesian Monte Carlo Markov Chain (MCMC) approach, which readily allows the development of complex random effects models [29]. Age and sex were retained in all models during the model identification process. Variables were added to the models in a forward stepwise fashion, comparing the statistical fits of alternative (nested and un-nested) models using both the residual deviance of the models and the Deviance Information Criteria (DIC; where a lower value indicates a better compromise between model fit and parsimony). A hierarchical approach was adopted when entering collinear predictor variables, whereby distal determinants (such as relative socio-economic status) are included prior to more proximal determinants (such as crowding and sanitation) [30]. Detailed descriptions of the Bayesian hierarchical models and the process of model assessment are described in Appendix S1.

Spatial heterogeneity (or structure) refers to the spatially non-random distribution of infection across the study region, such that an individual's risk of infection may be more similar to those living close to them that those living farther away. Such spatial clustering is not necessarily synonymous with clustering *within* households, because, whilst individuals in the same household may have more similar risk than individuals in different households, household-level risk may or may not be spatially autocorrelated. In order to examine the spatial structure of co-infection with *N. americanus* and *S. mansoni* at the household level, semi-variograms were generated using the R module *GeoR* on the basis of household prevalence of mono-infection with *N. americanus* and *S. mansoni* and co-infection with both parasites. Before variography, the data was de-trended by regressing against longitude and latitude, in order to remove large-scale spatial trends.

Semi-variograms present the semi-variance (i.e. half the mean squared difference) of pairs of observations that are separated by the same distance; thus, describing how similar observations are at different spatial distances [31]. If there is spatial autocorrelation in the data semi-variance increases with separation distance; levelling out of the semi-variogram indicates the distance beyond which spatial autocorrelation ceases to occur. When the semi-variogram appears to show little or no spatial autocorrelation, Monte Carlo envelopes (computed from random permutations of the residuals from random permutations of the data holding the corresponding locations fixed) can be used to assess more formally whether the data are compatible with spatial structure, under the assumption of no correlation [32],[33]. If the variogram plot falls within the envelope, there is no evidence of spatial autocorrelation at that distance.

## Results

### Infection patterns

Of the 1687 residents of the mapped households, 1539 individuals provided stool samples. Sixteen households (59 residents) in the far south-east of the study site were excluded from analysis because cloud-free satellite data were not available. Socio-economic data were unavailable for a further 275 individuals, who mainly lived in the urban municipality. As such, 1208 individuals living in 275 households had complete data. Households with GPS positions less than 10 m apart were treated as a single spatial unit, providing data for 230 locations for spatial analysis, the largest of which had 16 residents.

The majority of individuals were infected with helminths: 71.1% were infected with *N. americanus*, 50.3% had *S. mansoni* and 41.0% of individuals were co-infected with both helminths (co-infection). 30.1% were infected with only *N. americanus*, and only 9.4% of individuals were infected with only *S. mansoni*. The prevalence of co-infection was significantly higher among males than females (p<0.001) and increased significantly with increasing age, peaking among persons aged 20–59 years (p<0.001) (Table 1). The occurrence of co-infection also varied considerably by household, with prevalence varying from 0–100% (interquartile range: 0–67%); in 13.5% of households, all residents were co-infected with *N. americanus* and *S. mansoni*.

**Table 1:** Results of univariable logistic regression models (baseline outcome = uninfected).

	*N. americanus* ln[epg+1]			*S. mansoni* ln[epg+1]		
	*n = 1332*			*n = 1340*		
	*n*	Coefficient	*P* value	*n*	Coefficient	*P* value
**Demography**						
Sex						
Female vs. male	676	−0.61	<0.001	679	−0.15	0.28
Age						
*β* -Age (yrs) a	-	−0.013	0.006	-	−0.09	<0.001
[*β* - Age (yrs)]^2^ a	-	−0.0001	0.20	-	0.001	<0.001
Adult (20–59 yrs)	480	0	-	484	0	-
<5 years	175	−2.15	<0.001	176	−1.71	<0.001
5–9 years	211	−0.15	<0.001	176	−1.71	<0.001
10–19 years	327	0.44	0.04	328	0.46	0.04
60+ years	139	−0.06	0.85	139	−1.05	<0.001
**Socioeconomic status (vs. poorest)**						
More poor	143	0.06	0.88	144	0.65	0.19
Median	258	−0.53	0.15	257	0.93	0.005
Less poor	282	−1.34	<0.001	283	0.96	<0.001
Least poor	225	−2.42	<0.001	225	0.83	0.01
**Household characteristics**						
No toilet vs. toilet	790	2.15	<0.001	718	−0.35	0.13
Crowded household vs. uncrowded	674	1.20	<0.001	681	−0.19	0.41
Mud floor vs concrete/tiled floor	594	1.78	<0.001	602	−0.09	0.69
**Geographical Environment**						
Low density vs. high density housing	770	1.77	<0.001	778	−0.83	<0.001
Eastern vs. western watershed	846	−0.60	0.04	847	2.19	<0.001
*β* - ndvi a	-	−4.54	<0.001	-	0.93	0.14
*β* - dem a	-	−0.008	<0.001	-	0.005	<0.001

a (*β*-X) represents the effect associated with a 1-unit deviation from the mean level of the covariate X in the overall sample.

### Spatial heterogeneity

**Figure 2:**
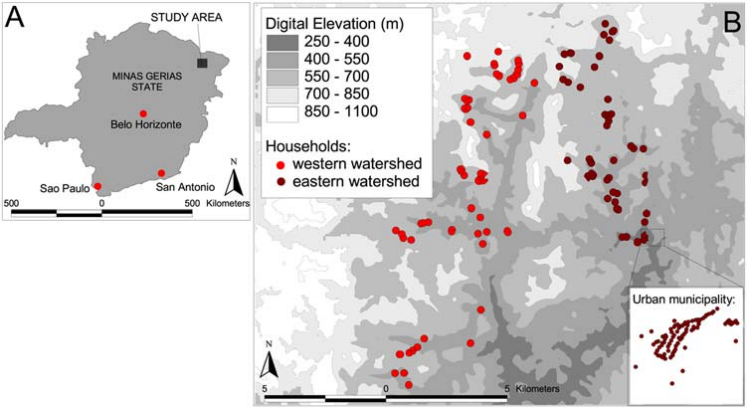
Household-level prevalence of helminth infection. Household prevalence of A egg-positive *N. americanus* mono-infection B egg-positive *S. mansoni* mono-infection and C *N. americanus* -*S. mansoni* co-infection among 1208 individuals living in 275 households. Values were calculated for an area of 200 m around each household and assigned to Thiessen polygons drawn on the basis of household positions.

**Figure 3:**
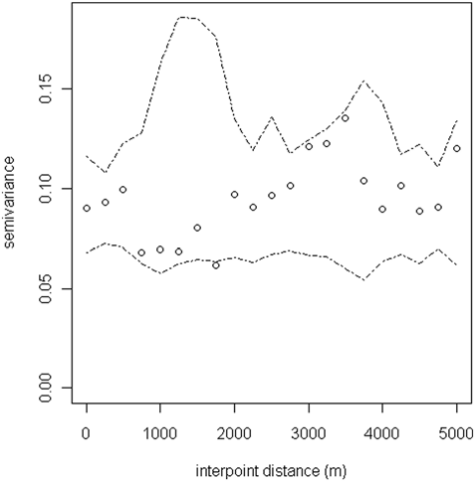
Spatial autocorrelation of infection status. Omni-directional semi-variogram for (de-trended) *N. americanus*-*S. mansoni* co-infection at the household level. Lag distance 250 m.

Figure 2 shows the spatial distribution of mono-infection with either *N. americanus* or *S. mansoni* or co-infection with both. The highest frequencies of co-infection were observed in the east of the study area, with an overall prevalence of 86.3% compared to 13.7% in the western watershed. To investigate the global spatial structure of infection patterns semi-variograms were estimated on the basis of household prevalence of mono-infection with *N. americanus* and *S. mansoni* and co-infection with both parasites. After removal of the large-scale spatial trend (by regressing against longitude and latitude) there was an apparent lack of any spatial structure for both *N. americanus* and *S. mansoni* mono-infection across all separation distances (not shown). Likewise, the semi-variogram for co-infection provides no evidence of spatial dependency, indicating that once the large-scale trends were removed there was no general spatial structure in the distribution of co-infection (Figure 3).

### Risk factors

Relative frequencies of household and environmental factors are shown in Table 1 according to infection status. Unadjusted results from fixed effects multinomial analyses showed that characteristics associated with lower socioeconomic status (SES index, toilet facilities, household crowding, flooring material), and residential environment (living in the eastern watershed, in more densely populated areas, or in areas with less vegetation) were significantly associated with both mono-infections and with co-infection (*p*<0.01).

Posterior estimates from the adjusted analysis using a hierarchical Bayesian multinomial mixed effects model confirm that the risk of co-infection relative to being uninfected was highest among males and adults aged 20–59 years (Table 2). There was also evidence of an increased risk of co-infection among individuals resident in households with a lower socio-economic index and in overcrowded households. After accounting for relative socio-economic status, toilet facilities and flooring material were no longer significant due to considerable co-linearity between these variables. Individuals living in the eastern watershed were 6.9 times more likely to harbour a co-infection than those living in the western watershed, while those living in areas with less vegetation cover (NDVI<0.2) were at reduced risk of co-infection. Associations between risk of infection and characteristics relating to lower socioeconomic status were observed for mono-infection with *N. americanus*, but not for *S. mansoni* mono-infection, while residential environment was associated with both mono-infections.

**Table 2:** Results of final Bayesian hierarchical multinomial model.

	*N. americanus* ln[epg+1]			*S. mansoni* ln[epg+1]		
	*n = 1332*			*n = 1340*		
	*n*	Coefficient	*P* value	*n*	Coefficient	*P* value
**Demography**						
Sex						
Female	1		1	-	1	-
Male	**2.22**	(1.48–.36)	1.13	(0.66–1.89)	**2.30**	(1.54–3.49)
Age						
Adult (20–59 yrs)	1	-	1	-	1	-
<5 years	**0.24**	(0.13–0.44)	**0.24**	(0.09–0.59)	**0.04**	(0.02–0.08)
5–9 years	**2.00**	(1.08–3.74)	1.93	(0.88–4.22)	**0.33**	(0.17–0.64)
10–19 years	**2.44**	(1.34–4.45)	**3.40**	(1.65–6.99)	1.50	(0.85–2.71)
60+ years	**1.57**	(0.80–3.08)	0.60	(0.23–1.45)	**0.45**	(0.22–0.90)
**Household characteristics**						
Socio-economic status						
Poor	1	-	1	-	1	-
Least poor	**0.40**	(0.23–0.68)	1.11	(0.57–2.10)	**0.34**	(0.18–0.62)
Household crowding						
1+ rooms / person	1	-	1	-	1	-
<1 rooms / person	**1.85**	(1.07–3.23)	0.81	(0.40–1.66)	**2.35**	(1.25–4.25)
**Location characteristics**						
Watershed						
East	1	-	1	-	1	-
West	**0.37**	(0.21–0.65)	**5.05**	(2.12–13.37)	**6.86**	(3.42–14.28)
NDVI						
0.2 and over	1	-	1	-	1	-
<0.2	**0.40**	(0.21–0.71)	1.06	(0.55–2.13)	**0.39**	(0.20–0.74)
**Random effect**						
Household-level σ^2^ (*u_i_*)	0.99	(0.4–1.9)	1.27	(0.4–2.8)	2.54	(1.4–4.1)

*: ROR = relative odds ratio; relative odds of the outcome vs. the baseline outcome (uninfected) for those in the exposed group compared with those who are not. RORs presented in bold are significant at the 5% level as indicated by the 95% BCI (Bayesian Credible Interval).

### Role of risk factors in household clustering

There was significant household clustering for all outcomes, as indicated by estimates for the household level random effects; the highest degree of unexplained household-level variation was observed for co-infection. Household-level variance was substantially higher when household and environmental risk factors were excluded from the model (Table 3). Whilst 40% of household-level variation could be explained by relative socio-economic status and household crowding for *N. americanus* mono-infection (i.e. inclusion of these covariates reduced the household-level variance parameter *u_i_* by 40%), substantially less household heterogeneity was explained by these factors for *S.mansoni* mono-infection (8%) and co-infection (10%). In contrast, environmental factors (living in the eastern watershed and areas with low NDVI) explained 36% of household-level variation in *N. americanus* mono-infection, 45% in *S.mansoni* mono-infection but only 19% in co-infection. Household and environmental factors jointly explained 54% of household variation in *N. americanus* mono-infection, but only 39.5% for *S. mansoni* mono-infection and 31.9% for co-infection.

**Table 3:** Comparison of household-level variance for models containing (i) only individual, (ii) individual and household, (iii) individual and environmental covariates, and (iv) the ‘full model’.

	Household-level σ^2^ (*u_i_*)		
	*N. americanus* mono-infection	*S. mansoni* mono-infection	*N. americanus*-*S. mansoni* co-infection
Model 1 (Age and sex)	2.15	2.10	3.73
Model2 (+household characteristics)	1.29	1.94	3.35
Model 3 (+location characteristics)	1.37	1.15	3.01
Full model (+household and location characteristics)	0.99	1.27	2.54

Fixed effect estimates vary little between models and so are not shown.

## Discussion

We employed a combination of spatial statistics and hierarchical multinomial modelling to investigate spatial patterns and household and environmental factors influencing occurrence of mono- and co-infection by the helminths *N. americanus* and *S. mansoni*. Our multi-level approach has the advantage of taking into account household clustering of infection, a commonly observed feature of helminth epidemiology [24],[34],[35]. The results suggest that, in addition to age and sex, characteristics associated with lower socioeconomic status (relative socio-economic status, household crowding) and residential environment (living in the eastern watershed or in areas with less vegetation) were significantly associated with the risk of co-infection relative to being uninfected with either species. Risk factors for co-infection reflected those associated with mono-infection, with no identified risk factors specific to co-infection.

The results presented in Table 2 and Figure 2 provide strong evidence of household clustering of co-infection with *N. americanus* and *S. mansoni*. While household clustering of single helminth infections is well-documented [24],[36],[37], the factors potentially responsible for such patterns remain less clear. Our observation that co-infection with *N. americanus* and *S. mansoni* is more common in households with lower relative socio-economic status is consistent with a study among schoolchildren in rural Cote d'Ivoire, which investigated school-level patterns in co-infection with *N. americanus* and *S. mansoni* [20]. Together, these studies suggest that socio-economic status influences the risk of co-infection at both household and local levels. The mechanisms through which socio-economic status influences infection risk are likely to reflect exposure-related factors, including poor hygienic behaviour, lack of clean water and inadequate sanitation, household construction (e.g. cement or dirt floors) and access to effective anthelmintics [38]–[41]. The increased risk of co-infection in households located in areas of higher NDVI (indicative of increased humidity and soil moisture) and in overcrowded households are consistent with previous studies reporting associations between hookworm and NDVI [42] and between helminth infection and overcrowding [43]–[45].

Our data demonstrated a dominant spatial trend (NE-SW) in household prevalence of co-infection (Figure 2), but there was little evidence of a second order spatial structure once this has been removed by regressing the data against latitude and longitude and plotting a semi-variogram of the model residuals (Figure 3). We suggest therefore that previous observations of small-scale spatial structure [42] probably reflect a combination of spatial variation in household characteristics and environmental risk factors. The absence of second order spatial structure is likely to reflect the high spatial resolution of the study, and it is plausible that in larger study areas and in areas with different eco-epidemiological and socio-economic characteristics, clearer spatial patterns may emerge; it would therefore be useful to investigate these issues in different epidemiological settings and at varying spatial scales.

The dominant NE-SW trend in co-infection observed reflects the distribution of *S. mansoni* rather than *N. americanus*, which is more homogeneously distributed across the study area. The high prevalence of *S. mansoni* in the east of the study region is likely to reflect the increased infectivity of water bodies in this area. It has been frequently demonstrated that within communities high intensity *Schistosoma* infections can be found clustered around water bodies such as rivers and lakes [36],[46],[47]. A limitation of our study is the lack of information regarding infectious water sources. We were unable to find recent and geo-referenced topographic maps from the area under investigation at the desired scale and quality, and it was not possible to delineate water-bodies from our remotely sensed images. Household water sources in this region of Brazil are typically small and private to each household, thus making them difficult to identify; this is reflected by the absence of large-scale spatial correlation between locations, suggesting that there are few large transmission sites (such as large, communal water sources) shared by many widely spaced households.

In terms of extrapolation to other settings, Americaninhas is representative of areas of rural northeast Minas Gerias state where helminth infections are highly endemic. Factors which may vary in other settings include contrasting socio-economic and environmental conditions, giving rise to different patterns and risk factors. However, our adopted analytical approach provides a robust methodology to further investigate the epidemiology of polyparasitism in other settings. A final potential limitation of our study, which applies to all multinomial analyses, is the assumption of Independent Irrelevant Alternatives (IIA), which essentially states that the risk associated with each outcome will not change if a new outcome is introduced. However, we believe that this analysis should not be restricted by IIA because our four choices exhaust the available responses (there are no other possible outcomes involving these two infections) [48].

A key finding of our study was that household and environmental risk factors could only account for an estimated 32% of variation between households in the risk of co-infection. Furthermore, unexplained household-level variation of co-infection with *N. americanus* and *S. mansoni* was considerably greater than for mono-infection with either *N. americanus* or *S. mansoni*. Although this may simply be a reflection of additive household variation associated with each species, it may alternatively be indicative of household factors specifically influencing risk of co-infection. For example, extrinsic factors such as location and infectivity of household water-sources [36],[39], or hygiene behaviours, health knowledge and water-contact patterns shared by members of the same households [49],[50] may influence exposure to both infections.

Unaccounted household-level variability may alternatively be explained by intrinsic host-related factors such as genetics [51],[52], nutrition [53], immune response [54],[55] or concomitant infection with other parasites [56]. Despite an increasing number of studies suggesting a genetic component to variation in intensity of helminth infection, the relative importance of host genetics and exposure remain unclear and vary considerably between the settings studied (reviewed in [57] and [58]). For example, in Zimbabwe, 37% of the total variation in *N. americanus* infection intensity was attributed to genetic factors [59], while in Brazil genetic factors only explained 21% of total variation in *S. mansoni* infection intensity [60]. To our knowledge, the genetic component of helminth co-infection has been investigated in only one study, conducted among residents of a rural community in Jiangxi Province, China [61]. The results of this study suggested that the risk of infection with multiple helminth species (*Schistosoma. japonicum*, *Trichuris trichuria* and *A. lumbricoides*) was in part explained by both genetic (16% of total variation) and household (9%) components. This was however a post-treatment study setting, hindering interpretation of results and preventing analysis of infection intensity.

Household clustering of helminth infection may also be influenced by genetic heterogeneity in the parasite population. Numerous molecular studies have revealed allelic and nucleotide diversity in the genomes of human helminth parasite populations [62],[63], with genetic variation occurring even among parasites sampled at very fine spatial scales [64]. As such, similarities in infection status within households may be in part due to parasite-relatedness, rather than host-relatedness [18]. However, separating the effects of host and parasite genetics on the variation in helminth infection remains a formidable task.

Whilst studies at micro-epidemiological scales are less useful for mapping and prediction of the distribution of co-infection, they are valuable in identifying why certain individuals within communities are at increased risk of multiple helminth infection, and as such have an increased risk of morbidity [15]. The balance between exposure and host-related factors such as genetics, nutrition, or the immune response as determinants of infection remains one of the most fundamental questions in parasite epidemiology and is a critical element in the rational development of control approaches [18]. The results presented here demonstrate considerable household clustering of co-infection, which could not be explained by a number of micro-climatic, socio-economic and other exposure-related factors. This further emphasises the role of the household in the heterogeneous distribution of helminth co-infection in human communities, pointing to the involvement of behavioural or genetic factors. Previous studies of household and familial clustering of single-species helminth infection have reached conflicting conclusions [34],[35],[65],[66]; few have simultaneously estimated the influence of both genetic and environmental factors [51],[60],[67], and only one has quantified influences on co-infection [61]. Future work is clearly needed to untangle the role of host factors such as genetic relatedness from household and environmental determinants of infection if we are to fully understand the basic epidemiology of human helminth infection at a community level.

## Supporting Information

Appendix S1 (0.03 MB PDF)

## References

[1] Howard SC, Donnelly CA, Kabatereine NB, Ratard RC, Brooker S (2002). Spatial and intensity-dependent variations in associations between multiple species helminth infections.. Acta Tropica.

[2] Drake LJ, Bundy DA (2001). Multiple helminth infections in children: impact and control.. Parasitology.

[3] Brooker S, Miguel EA, Moulin S, Luoba AI, Bundy DA (2000). Epidemiology of single and multiple species of helminth infections among school children in Busia District, Kenya.. East African Medicine Journal.

[4] Chamone M, Marques CA, Atuncar GS, Pereira AL, Pereira LH (1990). Are there interactions between schistosomes and intestinal nematodes?. Transactions of the Royal Society of Tropical Medicine and Hygiene.

[5] Booth M, Bundy DA, Albonico M, Chwaya HM, Alawi KS (1998). Associations among multiple geohelminth species infections in schoolchildren from Pemba Island.. Parasitology.

[6] Kightlinger LK, Seed JR, Kightlinger MB (1995). The epidemiology of Ascaris lumbricoides, Trichuris trichiura, and hookworm in children in the Ranomafana rainforest, Madagascar.. Journal of Parasitology.

[7] Haswell-Elkins MR, Elkins DB, Anderson RM (1987). Evidence for predisposition in humans to infection with Ascaris, hookworm, Enterobius and Trichuris in a South Indian fishing community.. Parasitology.

[8] Holland CV, Asaolu SO, Crompton DW, Stoddart RC, Macdonald R (1989). The epidemiology of Ascaris lumbricoides and other soil-transmitted helminths in primary school children from Ile-Ife, Nigeria.. Parasitology.

[9] Ferreira CS, Ferreira MU, Nogueira MR (1994). The prevalence of infection by intestinal parasites in an urban slum in Sao Paulo, Brazil.. Journal of Tropical Medicine and Hygiene.

[10] Needham C, Kim HT, Hoa NV, Cong LD, Michael E (1998). Epidemiology of soil-transmitted nematode infections in Ha Nam Province, Vietnam.. Tropical Medicine & International Health.

[11] Faulkner H, Turner J, Behnke J, Kamgno J, Rowlinson MC (2005). Associations between filarial and gastrointestinal nematodes.. Transactions of the Royal Society of Tropical Medicine and Hygiene.

[12] Brito LL, Barreto ML, Silva Rde C, Assis AM, Reis MG (2006). Moderate- and Low-Intensity Co-Infections by Intestinal Helminths and Schistosoma Mansoni, Dietary Iron Intake, and Anemia in Brazilian Children.. American Journal of Tropical Medicine & Hygiene.

[13] Pullan R, Brooker S (2008). The health impact of polyparasitism in humans: are we under-estimating the burden of parasitic diseases?. Parasitology.

[14] Ezeamama AE, Friedman JF, Olveda RM, Acosta LP, Kurtis JD (2005). Functional significance of low-intensity polyparasite helminth infections in anemia.. Journal of Infectious Disease.

[15] Brooker S, Akhwale WS, Pullan R, Estambale B, Clarke S (2007). Epidemiology of *Plasmodium*-Helminth coinfeciton in Africa: potential impact on anaemia and prospects for combining control.. American Journal of Tropical Medicine & Hygiene.

[16] Saldiva SR, Silveira AS, Philippi ST, Torres DM, Mangini AC (1999). Ascaris-Trichuris association and malnutrition in Brazilian children.. Paediatric and Perinatal Epidemiology.

[17] Bundy DAP, Chandiwana SK, Homeida MMA, Yoon S, Mott KE (1991). The epidemiological implications of a multiple-infection approach to the control of human helminth infections.. Transactions of the Royal Society of Tropical Medicine and Hygiene.

[18] Bundy DA, Medley GF (1992). Immuno-epidemiology of human geohelminthiasis: ecological and immunological determinants of worm burden.. Parasitology.

[19] Warren KS (1973). Regulation of the prevalence and intensity of schistosomiasis in man: immunology or ecology?. J Infect Dis.

[20] Raso G, Vounatsou P, Singer BH, N'Goran EK, Tanner M (2006). An integrated approach for risk profiling and spatial prediction of Schistosoma mansoni-hookworm coinfection.. Proc Natl Acad Sci U S A.

[21] Fleming FM, Brooker S, Geiger SM, Caldas IR, Correa-Oliveira R (2006). Synergistic associations between hookworm and other helminth species in a rural community in Brazil.. Tropical Medicine & International Health.

[22] Hotez PJ, Brooker S, Bethony JM, Bottazzi ME, Loukas A (2004). Hookworm infection.. New England Journal of Medicine.

[23] Friedman JF, Kanzaria HK, McGarvey ST (2005). Human schistosomiasis and anemia: the relationship and potential mechanisms.. Trends in Parasitology.

[24] Brooker S, Alexander N, Geiger S, Moyeed RA, Stander J (2006). Contrasting patterns in the small-scale heterogeneity of human helminth infections in urban and rural environments in Brazil.. Int J Parasitol.

[25] Brooker S, Jardim-Botelho A, Quinnell RJ, Geiger SM, Caldas IR (2007). Age-related changes in hookworm infection, anaemia and iron deficiency in an area of high Necator americanus hookworm transmission in south-eastern Brazil.. Trans R Soc Trop Med Hyg.

[26] de Gruijter JM, van Lieshout L, Gasser RB, Verweij JJ, Brienen EA (2005). Polymerase chain reaction-based differential diagnosis of Ancylostoma duodenale and Necator americanus infections in humans in northern Ghana.. Tropical Medicine & International Health.

[27] Tatem AJ, Goetz SJ, Hay SI (2004). Terra and Aqua: new data for epidemiology and public health.. International Journal of Applied Earth Observation and Geoinformation.

[28] Filmer D, Pritchett LH (2001). Estimating wealth effects without expenditure data–or tears: an application to educational enrollments in states of India.. Demography.

[29] Gilks WR, Richardson S, Spiegelhalter DJ (1996). Markov Chain Monte Carlo in Practice.

[30] Victoria CG, Huttly SR, Fuchs SC, Olinto MT (1997). The role of conceptual frameworks in epidemiological analysis: a hierarchical approach.. Int J Epidemiol.

[31] Chiles J-P, Delfiner P (1999). Geostatistics.

[32] Diggle PJ, Ribeiro PJ, York SN (2007). An overview of model based geostatistics.. Model Based Geostatistics.

[33] Ribeiro PJ, Christensen OF, Diggle PJ (2003). geoR and geoRglm: Software for Model-Based Geostatistics.. 3rd International Workshop on Distributed Statistical Computing.

[34] Forrester JE, Scott ME, Bundy DA, Golden MH (1988). Clustering of Ascaris lumbricoides and Trichuris trichiura infections within households.. Trans R Soc Trop Med Hyg.

[35] Behnke JM, De Clercq D, Sacko M, Gilbert FS, Ouattara DB (2000). The epidemiology of human hookworm infections in the southern region of Mali.. Trop Med Int Health.

[36] Clennon JA, King CH, Muchiri EM, Karuiki HC, Ouma JH (2004). Spatial patterns of urinary schistosomiasis infection in a highly endemic area of coastal Kenya.. am J Trop Med Hyg.

[37] Shapiro AE, Tukahebwa EM, Kasten J, Clarke SE, Magnussen P (2005). Epidemiology of helminth infections and their relationship to clinical malaria in southwest Uganda.. Trans R Soc Trop Med Hyg.

[38] Holland CV, Taren DL, Crompton DW, Nesheim MC, Sanjur D (1988). Intestinal helminthiases in relation to the socioeconomic environment of Panamanian children.. Soc Sci Med.

[39] Bethony J, Williams JT, Kloos H, Blangero J, Alves-Fraga L (2001). Exposure to Schistosoma mansoni infection in a rural area in Brazil. II: household risk factors.. Trop Med Int Health.

[40] Raso G, Utzinger J, Silue KD, Ouattara A, Yapi A (2005). Disparities in parasitic infections, perceived ill health and access to health care among poorer and less poor schoolchildren of rural Côte d'Ivoire.. Trop Med Int Health.

[41] Hotez P (2007). Hookworm and poverty.. Ann N Y Acad Sci.

[42] Saathoff E, Olsen A, Sharp B, Kvalsvig JD, Appleton CC (2005). Ecologic covariates of hookworm infection and reinfection in rural Kwazulu-natal/south Africa: a geographic information system-based study.. Am J Trop Med Hyg.

[43] Haswell-Elkins M, Elkins D, Anderson RM (1989). The influence of individual, social group and household factors on the distribution of Ascaris lumbricoides within a community and implications for control strategies..

[44] Narain K, Rajguru SK, Mahanta J (2000). Prevalence of Trichuris trichiura in relation to socio-economic and behavioural determinants of exposure to infection in rural Assam.. Indian Journal of Medical Research.

[45] Olsen A, Samuelsen H, Onyango-Ouma W (2001). A study of risk factors for intestinal helminth infections using epidemiological and anthropological approaches.. J Biosoc Sci.

[46] Clennon JA, Mungai P, Muchiri E, King CH, Kitron U (2006). Spatial and temporal variations in local transmission of *Schistosoma haematobium* in Msambweni, Kenya.. Am J Trop Med Hyg.

[47] Pinot de Moira A, Fulford AJ, Kabatereine NB, Kazibwe F, Ouma JH (2007). Microgeographical and tribal variations in water contact and Schistosoma mansoni exposure within a Ugandan fishing community.. Trop Med Int Health.

[48] Borooah VK (2002). Logit and Probit: ordered and Multinomial Models.

[49] Watts S, Khallaayoune K, Bensefia R, Laamrani H, Gryseels B (1998). The study of human behavior and schistosomiasis transmission in an irrigated area in Morocco.. Soc Sci Med.

[50] Bethony J, Williams JT, Brooker S, Gazzinelli A, Gazzinelli MF (2004). Exposure to Schistosoma mansoni infection in a rural area in Brazil. Part III: household aggregation of water-contact behaviour.. Trop Med Int Health.

[51] Williams-Blangero S, McGarvey ST, Subedi J, Wiest PM, Upadhayay RP (2002). Genetic component to susceptibility to Trichuris trichiura: evidence from two Asian populations.. Genet Epidemiol.

[52] Bethony J, Gazzinelli A, Lopes A, Pereira W, Alves-Oliveira L (2001). Genetic epidemiology of fecal egg excretion during Schistosoma mansoni infection in an endemic area in Minas Gerais, Brazil.. Mem Inst Oswaldo Cruz.

[53] Bundy DAP, Golden MH (1987). The impact of host nutrition on gastrointestinal helminth populations.. Parasitology.

[54] Loukas A, Constant SL, Bethony JM (2005). Immunobiology of hookworm infection.. FEMS Immunol Med Microbiol.

[55] Pearce EJ, MacDonald AS (2002). The immunobiology of schistosomiasis.. Nat Rev Immunol.

[56] Cox FE (2001). Concomitant infections, parasites and immune responses.. Parasitology.

[57] Quinnell RJ (2003). Genetics of susceptibility to human helminth infection.. Int J Parasitol.

[58] Bethony J, Quinell RJ (2007). Genetic epidemiology of human schistosomiasis in Brazil.. Acta Trop [epub ahead of print].

[59] Williams-Blangero S, Blangero J, Bradley M (1997). Quantitative genetic analysis of susceptibility to hookworm infection in a population from rural Zimbabwe.. Hum Biol.

[60] Bethony J, Williams JT, Blangero J, Kloos H, Gazzinelli A (2002). Additive host genetic factors influence fecal egg excretion rates during Schistosoma mansoni infection in a rural area in Brazil.. Am J Trop Med Hyg.

[61] Ellis MK, Raso G, Li Y-S, Rong Z, Chen H-G (2007). Familial aggregation of human susceptability to co-and multiple helminth infections in a population from the Poyang Lake region, China.. Int J Parasitol.

[62] Stohler RA, Curtis J, Minchella DJ (2005). A comparison of microsatellite polymorphism and heterozygosity among field and laboratory populations of Schistosoma mansoni.. Int J Parasitol.

[63] Curtis J, Sorensen RE, Minchella DJ (2002). Schistosome genetic diversity : the implications of population structure as detected with microsatellite markers.. Parasitology.

[64] Hawdon JM, Li T, Zhan B, Blouin MS (2001). Genetic structure of populations of the human hookworm, Necator americanus, in China.. Mol Ecol.

[65] Chan L, Bundy DA, Kan SP (1994). Genetic relatedness as a determinant of predisposition to Ascaris lumbricoides and Trichuris trichiura infection.. Parasitology.

[66] Chan L, Bundy DA, Kan SP (1994). Aggregation and predisposition to Ascaris lumbricoides and Trichuris trichiura at the familial level.. Trans R Soc Trop Med Hyg.

[67] Williams-Blangero S, Subedi J, Upadhayay RP, Manral DB, Rai DR (1999). Genetic analysis of susceptibility to infection with Ascaris lumbricoides.. Am J Trop Med Hyg.

