# Supplementary material for: Human Helminth Co-Infection: Analysis of Spatial Patterns and Risk Factors in a Brazilian Community
Source: PLoS Negl Trop Dis. 2008 Dec 23;2(12):e352. doi: 10.1371/journal.pntd.0000352 (PMC2602736; doi:10.1371/journal.pntd.0000352)
Supplement: Appendix S1 — (0.03 MB PDF) [file pntd.0000352.s001.pdf]

Supplementary Information 1

The Bayesian hierarchical models were in the form:

Y_{ijk} ~ $MN(1,p_{ij})$ where $p_{ij} = (p_{ij1}, p_{ij2}, p_{ij3}, p_{ij4})$,

$$\log\left(\frac{p_{ijk}}{p_{ij1}}\right) = \alpha_k + \sum \beta_{kn} X_{ij} + u_{ik}$$

where Y_{ijk} and p_{ijk} is the occurrence and probability of no infection (k=1), *N. americanus* mono-infection (k=2), *S. mansoni* mono-infection (k=3) and *N. americanus - S. mansoni* co-infection (k=4) for individual j in household i, α_k is the intercept for infection status k, and $\Sigma\ \beta_{kn}$ is a vector of n regression parameters corresponding to the set of n covariates (X_{ij}). The component u_{ik} is the unstructured heterogeneity at the household level for infection status k. Due to potential dependency between the binary outcomes, the random effects may be correlated; they were therefore modelled using a multivariate distribution as explained below.

Fixed regression coefficients were assigned diffuse priors functionally equivalent to a vague normal prior with large variance. Unstructured, household-level clustering was estimated using an exchangeable multivariate normal prior, ($u_{j2}\, u_{j3}\, u_{j4}$) ~ $MVN(0, \Omega^{-1})$, with covariance matrix Ω^{-1} . The diagonal element of this covariance matrix is equal to the variance, whilst the off-diagonals are correlation components between infection status groups. The variance components were assigned the weakly informative diffuse inverse Wishart prior $p(\Omega^{-1})$ ~ Wishart (p, Q); where p the scalar and Q is a prior estimate of Ω. The hyperpriors were assigned p = 3 and $Q = I$ where I is an identity matrix.

Model fitting used Markov Chain Monte Carlo simulation techniques. A burn-in of 1000 iterations was allowed, followed by 10,000 iterations where values for the intercept and coefficients were stored. Diagnostic tests for convergence of the stored variables were undertaken, including visual examination of history and density plots of the model parameters, and by computing Monte Carlo errors (MCE; if MCE/SD was less than 0.05, it was decided that sufficient iterations had been conducted): convergence was successfully achieved after 10,000 iterations. The chains were also examined for autocorrelation by visual examination of the inbuilt autocorrelation function of WinBUGS. Considerable autocorrelation was apparent for the intercept and random coefficients and it was decided to reduce autocorrelation by thinning subsequent sampling by storing every 20th iteration. Thus, for all models 110 000 iterations were run, with the first 10,000 discarded ('burn in') and sub-sampled every 20th observation, giving a final sample size of 5000 for which the model parameters were estimated.

For sensitivity analysis of fixed effects we used alternative normal priors with varying variances. In addition, we tested sensitivity to prior estimates of the covariance matrix Q. Model comparison was based on the Deviance Information Criteria (DIC).
